# Epitranscriptomics as a Candidate Universal Modulator of Dormancy Transitions

**DOI:** 10.1002/ece3.73007

**Published:** 2026-02-03

**Authors:** Ehsan Pashay Ahi

**Affiliations:** ^1^ Organismal and Evolutionary Biology Research Programme, Faculty of Biological and Environmental Sciences University of Helsinki Helsinki Finland

**Keywords:** dormancy, eco‐evolutionary adaptation, epitranscriptomics, N6‐methyladenosine (m^6^A), RNA chemical modifications

## Abstract

Dormancy has been widely recognized as an evolutionarily conserved strategy that enables cells and organisms to endure environmental stress, resource scarcity, or developmental arrest. While transcriptional regulation has been extensively studied in this context, increasing attention is being directed toward post‐transcriptional mechanisms that allow rapid and energy‐efficient control of gene expression. Among these, epitranscriptomic modifications, chemical marks added to RNA, have emerged as dynamic and reversible regulators of mRNA fate. In this perspective, it is proposed that RNA modifications can play a central role in establishing and maintaining dormancy across diverse biological systems. Evidence from plant seeds, microbial persisters, stem cells, and dormant cancer cells suggests that specific RNA marks, such as N6‐methyladenosine (m^6^A), influence mRNA stability, translation, and localization in a context‐dependent manner. It is argued that these modifications serve as a molecular interface between environmental signals and cellular responses, fine‐tuning the transition between active and paused states. This article presents a unifying model, grounded in epitranscriptomics, in which RNA modifications modulate entry into, maintenance of, and exit from dormancy across taxa by tuning mRNA stability, translation, and localization—an underexplored regulatory layer in inactive states—and highlights key mechanistic insights, evolutionary parallels, and outstanding questions at the intersection of RNA regulation and cellular dormancy.

## Graphical abstract

Dormancy is presented as a conserved, reversible survival program in which epitranscriptomic RNA modifications are proposed to provide a rapid, energy‐efficient layer that establishes, maintains, and terminates the state by modulating mRNA stability, translation, and localization. Evidence spanning seeds, microbial persisters, stem cells, and dormant cancer cells is synthesized to advance a unifying model in which RNA marks are positioned as a sensor–effector interface linking environmental cues to dormancy decisions. Key mechanisms and evolutionary parallels are outlined, and outstanding questions are identified to guide future tests of this framework.

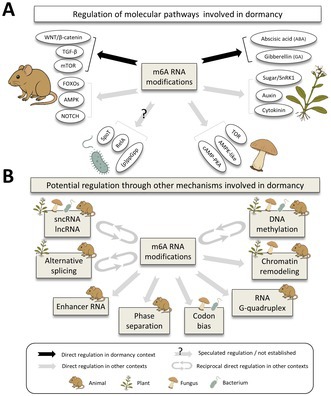

## 1 Introduction

Dormancy has been recognized as a widespread and evolutionarily conserved strategy that enables cells, tissues, and entire organisms to withstand periods of environmental or physiological stress (Miller et al. 2021; Webster and Lennon 2025). Across the tree of life, from unicellular bacteria to multicellular plants and mammals, dormancy has been employed as a temporally controlled mechanism that promotes survival during unfavorable or unpredictable conditions (Wilsterman et al. 2021; McDonald et al. 2024; Özgüldez and Bulut‐Karslioğlu 2024). Its prevalence across phylogenetically distant organisms has been interpreted as evidence of strong selective pressure favoring phenotypic plasticity and reversible growth arrest under stress (Wilsterman et al. 2021; Constant et al. 2023; Otake et al. 2024; Webster and Lennon 2025). In prokaryotes, dormancy has been observed in the form of spore formation or persister cell states, where replication is halted and metabolic activity is drastically reduced, allowing survival in the presence of antibiotics or immune responses (McDonald et al. 2024; Walker et al. 2024). In plants, seed dormancy has evolved as a developmental pause that is tightly regulated by environmental cues such as temperature, light, and moisture (Klupczyńska and Pawłowski 2021; Sajeev et al. 2024; Wang, Bu, et al. 2024). In animals, dormancy‐like states, including diapause in invertebrates and quiescence in adult stem cells, have been shown to underlie developmental timing and tissue regeneration (Wilsterman et al. 2021; Payton et al. 2024; Short et al. 2024; Saito et al. 2025; Yang, Woodruff, et al. 2025). In a pathological context, oncology increasingly attributes a dormant phenotype to disseminated tumor cells that evade chemotherapy and remain clinically undetectable for years before reactivation (Yang, Seo, et al. 2025).

Despite their varied contexts, all forms of dormancy are characterized by a shift in cellular priorities: from active proliferation or differentiation to survival and maintenance (Gomis and Gawrzak 2017; Pshennikova and Voronina 2022; Considine 2024). This transition is achieved through global suppression of biosynthetic processes, reduced transcriptional output, and highly selective translation of stress‐adaptive proteins (Buijs et al. 2020; Jobava et al. 2021; Tognacca and Botto 2021; Amissah et al. 2024; Koli and Shetty 2024). Such states are not only reversible but are often poised for rapid reactivation upon re‐exposure to permissive conditions (Pshennikova and Voronina 2022; Özgüldez and Bulut‐Karslioğlu 2024). Given the limitations of transcription‐based regulation in energy‐restricted environments (e.g., nutrient‐limited conditions), it has been hypothesized that post‐transcriptional control plays a central role in dormancy (Reynolds 2019; Craft et al. 2020; Luján‐Soto and Dinkova 2021; Tognacca and Botto 2021; Pi et al. 2022; Collignon et al. 2023). Recent studies have pointed to the significance of mRNA stabilization, selective translation, and RNA‐protein granule formation in sustaining the dormant state (Ignatov et al. 2015; Escalante and Gasch 2021; Collignon et al. 2023; Lorenzo‐Orts and Pauli 2024). These mechanisms allow cells to preserve transcripts for future use, degrade nonessential messages, or modulate translation rates in a transcript‐specific manner. However, the emerging field of epitranscriptomics has introduced an additional layer of regulation that may operate as a rapid and reversible switch during dormancy transitions (Shao et al. 2021; Collignon et al. 2023; Dhingra et al. 2023). Key terms used throughout the manuscript are defined in Box 1. Its recurrence across evolutionarily distant lineages suggests the existence of conserved molecular frameworks, among which RNA‐based regulation is increasingly considered to be fundamental (Table 1). A cross‐taxa logic is proposed in which RNA modifications tune mRNA stability, translation, and localization to support reversible dormancy transitions. At the same time, epitranscriptomic regulation specifically during inactive/dormant states remains comparatively underexplored, despite being well‐suited for rapid and energy‐efficient control of gene expression.

BOX 1 **Key concepts referred to in this article.**

**Table:** 

**Dormancy:** A reversible, energy‐conserving state in which cells or organisms halt growth and division to survive adverse conditions **Cellular quiescence:** A nonproliferative, reversible state often observed in stem cells and associated with dormancy **Metabolic rate depression (MRD):** A reversible, systemic reduction in energy turnover that suppresses ATP‐demanding processes while preserving viability; underpins hibernation, estivation, torpor, and diapause **Epitranscriptomics:** The study of chemical modifications on RNA molecules that influence their function, stability, and translation without altering nucleotide sequences **m6A (N6‐methyladenosine):** The most abundant internal mRNA modification in eukaryotes, regulating RNA metabolism through reader, writer, and eraser proteins **Stress granules:** Cytoplasmic aggregates of stalled translation initiation complexes that store mRNAs during stress or dormancy **P‐bodies:** Cytoplasmic sites for mRNA decay and storage, often active during translational repression in dormancy **Bet‐hedging:** An evolutionary strategy where phenotypic variability increases survival under fluctuating or unpredictable environments **Cap‐independent translation:** A mechanism of translation initiation not requiring the 5′ cap, often employed during stress or dormancy **Phase separation:** The formation of membraneless compartments (e.g., stress granules) through physicochemical interactions among proteins and RNAs **Pseudouridine (Ψ):** A common RNA modification that can alter RNA structure and translation, present in tRNAs, rRNAs, and some mRNAs **m5C (5‐methylcytosine):** A modification that can influence RNA export, localization, and stability, though its role in dormancy is still emerging

**TABLE 1:** Summary of dormancy types across organisms.

Dormancy type	Organisms	Mechanism type(s)	Signaling pathways	Approximate evolutionary origin (geological time)
Spore formation	Bacteria, cyanobacteria	Transcriptional shutdown, DNA compaction, metabolic arrest	Spo0A phosphorelay, sigma factor cascade, nutrient sensing	Precambrian
Cyst formation	Protists, nematodes	Translational repression, membrane remodeling	cAMP/PKA, MAPK, small GTPases	Precambrian–Cambrian
Anhydrobiosis	Tardigrades, rotifers, nematodes	Protein and RNA stabilization, metabolism suspension	Stress response pathways, ROS signaling	Precambrian–Cambrian
Diapause	Insects, fish, crustaceans	Hormonal signaling, translational and transcriptional repression	Insulin/FOXO, TOR, Juvenile hormone	Cambrian (Paleozoic)
Seed dormancy	Angiosperms	Hormonal control, mRNA storage, translational silencing	ABA/GA, DOG1 pathway, SnRK2	Silurian–Devonian (Paleozoic)
Bud dormancy	Trees and perennials	Hormonal cycling, chromatin remodeling	ABA, DAM gene regulatory network, cold signaling (CBF)	Carboniferous (Paleozoic)
Spore dormancy (Fungi)	Fungi	Transcriptional arrest, protein stabilization	Nutrient sensing, PKA, AMPK	Precambrian–Cambrian
Torpor/hibernation/aestivation	Mammals, amphibians, reptiles	Metabolic rate depression (MRD), thermoregulation	AMPK, insulin, mTOR, adrenergic signaling	Mesozoic–Cenozoic
Embryonic diapause (mammals)	Kangaroos, bears, some rodents	Uterine signals, blastocyst arrest, chromatin repression	mTOR, LIF/STAT3, estrogen receptor pathways	Cenozoic
Cancer cell dormancy	Vertebrates	Quiescence, immune evasion, metabolic reprogramming	p38 MAPK, ERK, autophagy, UPR, mTOR	Cenozoic

*Note:* Main regulatory mechanisms, key pathways, and approximate evolutionary origins (geological time labels). Evolutionary origin ranges are heuristic approximations based on the earliest documented phylogenetic distribution of each dormancy type across extant lineages (as summarized in synthesis literature), and are not intended to imply a single evolutionary origin event.

## 2 Dormancy‐Regulating Signaling Pathways Across Biological Kingdoms

**FIGURE 1:**
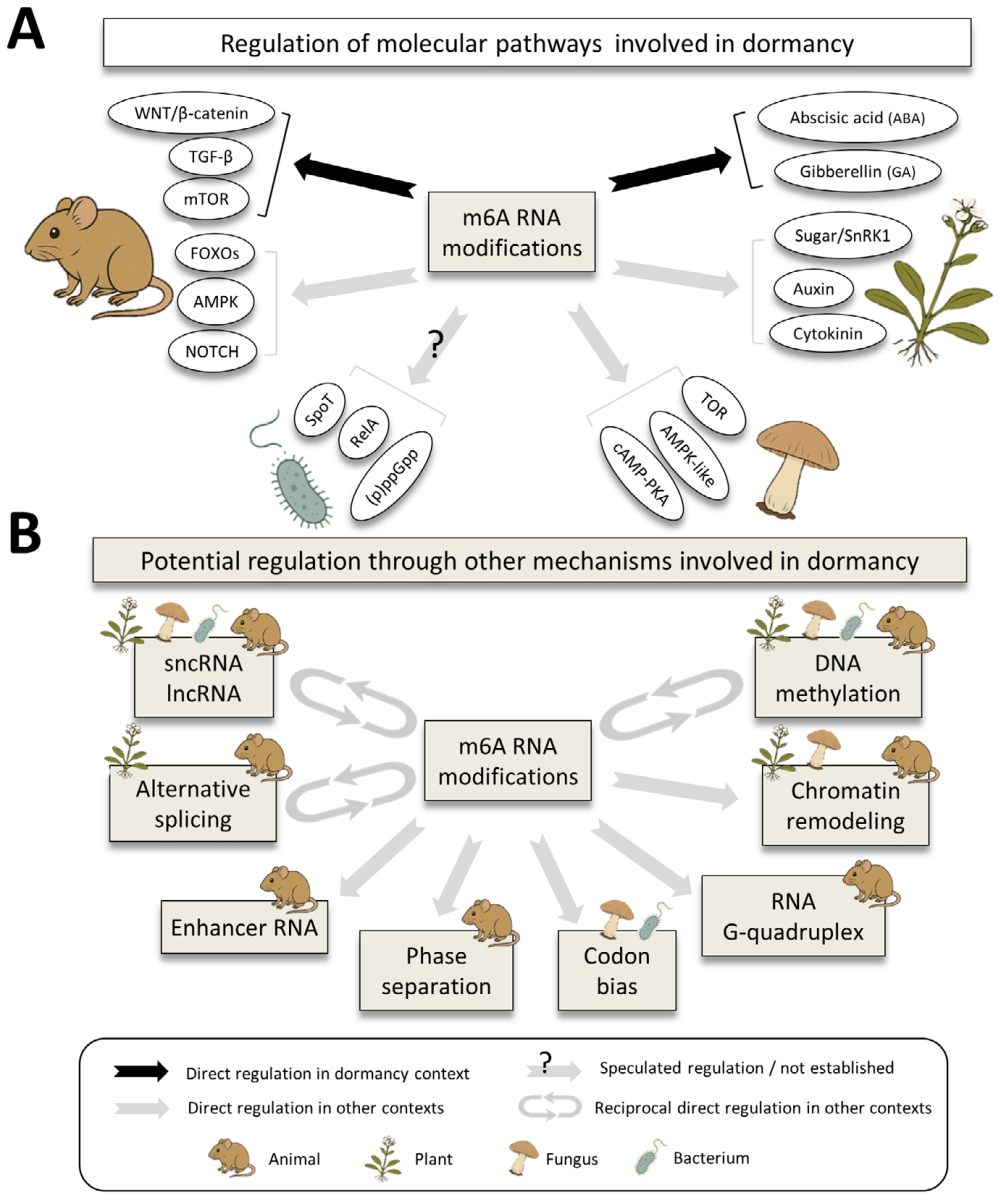
Roles of m6A in dormancy. (A) Key dormancy pathways across taxa and their links to m^6^A. Plant pathways: ABA/GA; sugar/SnRK1; auxin; cytokinin (Schaller et al. 2014; Tuan et al. 2018; Matilla 2020; Tang et al. 2021; Choudhary et al. 2022; Shen and Yu 2025); Animal pathways: mTOR/AMPK (Li, Deng, et al. 2021; Liu, Zuo, et al. 2023), FOXO (Lin et al. 2020; Li, Jin, et al. 2023; Liu et al. 2024); Wnt/β‐catenin (Li, Peng, et al. 2023; Zhang et al. 2023); TGF‐β (Fan et al. 2024; Zhang, Fu, et al. 2024); and Notch (Abravanel et al. 2015; Herrick et al. 2017). Fungal pathways: TOR/PKA/AMPK (Sun et al. 2019; Breeden and Tsukiyama 2022; Plank 2022). Bacterial pathways: RelA; SpoT; (p)ppGpp (McDonald et al. 2024; Abid et al. 2025). (B) How m^6^A may regulate dormancy via additional mechanisms. DNA methylation (Luján‐Soto and Dinkova 2021; Lieberman‐Lazarovich et al. 2022); chromatin remodeling (Swygert et al. 2019; Breeden and Tsukiyama 2022); RNA G‐quadruplex (Zuurbier et al. 2024); codon bias (Kanduc 2021; Small‐Saunders et al. 2024; Feng et al. 2025); phase separation (Xu et al. 2021; Liu, Zhu, and Zhao 2023); enhancer RNA (So et al. 2022; Tremblay et al. 2024); alternative splicing (Penfield et al. 2010; Li, Yan, et al. 2021; Zhu et al. 2023); sncRNA/lncRNA (Reynolds 2019). Icons indicate supporting evidence by taxon (animal, plant, fungus, bacterium).

Plant dormancy, particularly in seeds and buds, is governed primarily by the abscisic acid (ABA) and gibberellin (GA) signaling pathways (Tuan et al. 2018). ABA induces and maintains dormancy under stress by promoting desiccation tolerance and repressing growth‐related genes (Maia et al. 2014), while GA promotes dormancy release and germination by activating growth‐promoting gene expression (Ogawa et al. 2003). Importantly, it is the balance between ABA and GA (often expressed as the ABA/GA ratio) that integrates environmental cues and largely determines whether a seed remains dormant or proceeds toward germination (Zheng et al. 2015; Tuan et al. 2018). Sugar signaling, mediated through the SnRK1 kinase pathway, also plays a crucial role in energy sensing and metabolic adjustment during dormancy (Choudhary et al. 2022). Additional regulation comes from auxin and cytokinin signaling, which influence bud dormancy and reactivation (Schaller et al. 2014; Qiu et al. 2019; Matilla 2020). Recent research has demonstrated that m^6^A RNA methylation plays a key role in regulating these hormone pathways: m^6^A marks affect the stability and translation of ABA and GA pathway transcripts, thereby modulating the timing and sensitivity of dormancy induction and release (Tang et al. 2021; Amara et al. 2022; Wu et al. 2024; Shen and Yu 2025; Wang et al. 2025). For example, during seed germination (dormancy exit) in *Arabidopsis*, loss of the m^6^A demethylase ALKBH10B has been associated with ABA‐hypersensitive germination and m^6^A hypermethylation of ABA signaling transcripts including *PYR1*, *PYL7*, *PYL9*, *ABI1*, and *SnRK2.2* after ABA treatment, consistent with altered stability and/or utilization of these messages during the transition (Tang et al. 2021). In parallel, an m^6^A reader (ECT1) has been shown to promote GA‐linked germination by destabilizing *RGA1* (a GA pathway repressor) and by modulating PHYB/DAG2‐associated signaling outputs, providing a transcript‐level example for how m^6^A reading can tune hormone‐gated germination decisions (Li, Ma, et al. 2025). This indicates a functional epitranscriptomic layer fine‐tuning the plant's dormancy transitions (Figure 1A).

In animals, dormancy (often termed quiescence in stem cells or latency in cancer) involves a complex interplay of metabolic and stress‐related pathways (Dias et al. 2021; Özgüldez and Bulut‐Karslioğlu 2024). The mTOR and AMPK pathways are central (Pashay and House 2026): mTOR promotes growth and biosynthesis under favorable conditions (Bulut‐Karslioglu et al. 2016; Alhasan et al. 2021), while AMPK becomes activated during energy stress to conserve resources and promote dormancy (Rider 2015; Kadekar and Roy 2019; Kamata et al. 2023). FOXO transcription factors support dormancy through stress resistance and cell cycle arrest (van der Weijden et al. 2024), while pathways like TGF‐β, Notch, and Wnt/β‐catenin regulate stem cell quiescence and dormancy plasticity in cancer (Abravanel et al. 2015; Herrick et al. 2017; Prunier et al. 2019; Fan et al. 2020; Dias et al. 2021; van der Weijden and Bulut‐Karslioglu 2021). Increasingly, evidence highlights a significant role for m^6^A RNA methylation in modulating these pathways. For instance, m^6^A regulates mTOR and AMPK signaling by affecting the translation of key metabolic genes (Li, Deng, et al. 2021; Liu, Zuo, et al. 2023). m^6^A‐dependent regulation has been described for components of the LKB1–AMPK axis (linking energy stress to autophagy), and for PI4K pathway transcripts (*Pi4k2a/Pi4kb*) that influence Akt–mTOR signaling during exit from quiescence in muscle stem cells, providing mechanistic entry points connecting m^6^A to metabolic gating of dormancy‐like states (Li, Deng, et al. 2021; Liu, Zuo, et al. 2023). FOXO mRNAs are also subject to m^6^A‐dependent stabilization or decay, influencing stress adaptation (Lin et al. 2020; Li, Jin, et al. 2023; Liu et al. 2024). Specifically, METTL3‐installed m^6^A can promote YTHDF2‐mediated decay of *FOXO1* mRNA in some cellular settings, whereas m^6^A removal by ALKBH5 has been shown to increase *FOXO1* mRNA stability in chemoresistant cancer cells, illustrating how writer/eraser state and reader engagement can shift FOXO messages toward decay versus stabilization (Lin et al. 2020; Li, Jin, et al. 2023; Liu et al. 2024). In cancer cells, m^6^A modification of Wnt pathway transcripts modulates self‐renewal and exit from quiescence (Li, Peng, et al. 2023; Zhang et al. 2023). Similarly, TGF‐β pathway components are regulated by m^6^A‐dependent RNA decay or translational control, fine‐tuning cell cycle arrest and reactivation (Fan et al. 2024; Zhang, Fu, et al. 2024). These recent findings suggest that epitranscriptomic mechanisms are deeply embedded in the regulation of dormancy decisions in animal cells (Figure 1A).

Fungal dormancy is most commonly observed in spores and quiescent vegetative states, regulated primarily by nutrient‐responsive pathways like TOR, cAMP‐PKA, and AMPK‐like kinases (Sun et al. 2019; Plank 2022). When nutrients are scarce, TOR signaling is inhibited, prompting a shift from proliferation to dormancy; cAMP‐PKA signaling similarly balances growth and stasis. While epigenetic regulation in fungal dormancy is well‐established (Swygert et al. 2019; Breeden and Tsukiyama 2022; Opalek et al. 2023), epitranscriptomic regulation is an emerging field. Recent studies in *cerevisiae* have identified m^6^A modifications in transcripts related to metabolic adaptation and stress resistance, though specific pathway interactions are still being uncovered (Yadav and Rajasekharan 2017; Scutenaire et al. 2023). There is preliminary evidence that m^6^A affects mRNAs involved in the TOR and stress response pathways (Bodi et al. 2015; Ren, Tang, et al. 2022), likely influencing the timing of sporulation or quiescence (Figure 1A).

Bacterial dormancy, including sporulation, persistence, and latency, is regulated by unique prokaryotic pathways such as the stringent response (via (p)ppGpp), toxin–antitoxin systems, and two‐component regulatory systems (McDonald et al. 2024; Abid et al. 2025). These networks help cells survive antibiotic stress, nutrient deprivation, and immune evasion by shutting down transcription, translation, and replication. Unlike in eukaryotes, epitranscriptomic regulation in bacteria is still nascent, though it is gaining attention (Tan et al. 2024). Canonical eukaryotic m6A machinery (METTL3/METTL14, FTO/ALKBH5) has not been established in bacteria (Szydlo et al. 2025); instead, prokaryotic RNA modifications are installed by distinct enzyme families and frequently characterized on rRNA/tRNA (Pletnev et al. 2020), with growing reports on mRNA (Antoine et al. 2021; Tan et al. 2024). Some studies have identified bacterial RNA modifications, including m^6^A and m^5^C, in transcripts related to dormancy, persistence, and stress response (Vargas‐Blanco and Shell 2020; Antoine et al. 2021; Riquelme‐Barrios et al. 2025) (Figure 1A). At present, direct mechanistic coupling between canonical dormancy regulators (e.g., stringent–response components) and RNA methylation remains limited, and the available evidence is best described as preliminary (Pletnev et al. 2020; Yu et al. 2025). Notably, links between bacterial signaling/stress states and RNA methylation have been demonstrated in rRNA‐focused contexts; for example, c‐di‐GMP can inhibit rRNA methylation and impair ribosome assembly under antibiotic stress (Yu et al. 2025), indicating a plausible interface that remains to be tested explicitly in dormancy transitions. However, direct mechanistic crosstalk between specific dormancy pathways (e.g., RelA and SpoT‐mediated stringent response) and RNA methylation remains limited, and the available evidence is best described as preliminary rather than definitive.

## 3 Beyond Transcription: The Need for Post‐Transcriptional Control in Dormancy

Traditionally, dormancy has also been explored through the lens of gene expression regulation, with many studies focusing on stress‐responsive transcription factors, chromatin remodeling, and promoter‐level silencing. While such mechanisms have provided foundational insights, accumulating evidence suggests that transcriptional repression alone does not fully account for the dynamic, flexible, and energy‐efficient control required during dormancy. In many systems, dormancy has been shown to persist even when transcription is globally reduced, pointing to the existence of additional regulatory layers acting downstream of gene transcription.

A compelling need for post‐transcriptional control in dormancy arises from the metabolic constraints faced by cells in the dormant state (Storey and Storey 2004; Tognacca and Botto 2021; Dalziell et al. 2025). Transcription is an energy‐intensive process, and its global suppression under stress is both adaptive and necessary (Ramnanan et al. 2009; Storey and Storey 2012; Logan et al. 2019). However, survival during dormancy still requires the production of selective proteins involved in stress resistance, metabolic rewiring, and the maintenance of cellular architecture (Sajeev et al. 2019; Bezrukov et al. 2021; Lorenzo‐Orts et al. 2023). To resolve this paradox, many organisms rely on stored transcripts, which are preserved in a translationally silent state and selectively activated when needed (Bazin et al. 2011; Ignatov et al. 2015; Bai et al. 2020; Sano et al. 2020). This allows cells to maintain a minimal yet responsive proteome without initiating new transcription. For example, in plant seeds, bacteria and certain invertebrates, mRNAs critical for germination, sporulation, or developmental progression are localized to specific subcellular compartments and remain untranslated until favorable conditions return (Stuckas et al. 2014; Sano et al. 2020; Iwańska et al. 2024; Lorenzo‐Orts and Pauli 2024; Özgüldez and Bulut‐Karslioğlu 2024; Yang et al. 2024). In *Arabidopsis*, seed‐stored mRNAs have been shown to be selectively recruited to polysomes during germination (dormancy exit), whereas cap‐independent translation (including m6A‐enabled 5′UTR initiation and eIF4F‐independent translation) has been demonstrated under stress conditions and provides a concrete mechanistic example for how translation can proceed when cap‐dependent initiation is curtailed (Basbouss‐Serhal et al. 2015, 2017; Meyer et al. 2015; Coots et al. 2017; Bai et al. 2020). In stem cells and cancer cells, stress granules and P‐bodies serve as reservoirs for silenced mRNAs, whose fate is determined by post‐transcriptional cues rather than promoter activity (Lavut and Raveh 2012; Fefilova et al. 2022; Ren, Zhang, et al. 2022). These structures exemplify how dormancy involves dynamic mRNA regulation at the cytoplasmic level, where storage, decay, and translation are finely tuned within translational control hubs—stress granules and P‐bodies (see Box 1). Post‐transcriptional regulation has also been observed to interact with metabolic signaling pathways known to control dormancy, such as TOR (target of rapamycin) (Bulut‐Karslioglu et al. 2016; Yeh and Yong 2020; Alhasan et al. 2021) and AMPK pathways (Ramnanan et al. 2010; You et al. 2022). Through downstream targets such as 4E‐BP/eIF4E and S6K‐dependent translation components, mTORC1 can modulate cap‐dependent initiation, whereas AMPK can suppress global translation by inhibiting mTORC1 signaling and engaging elongation control (Ramnanan et al. 2009; Yeh and Yong 2020; You et al. 2022). In parallel, stress‐associated RNA‐binding proteins that scaffold translational control hubs (e.g., G3BP1/TIA‐1–like granule factors) can reshape mRNA triage between translation and storage (Fu and Zhuang 2020; Escalante and Gasch 2021). These mechanisms are well supported in stress‐induced growth‐arrest contexts and are proposed here as plausible mediators of selective translation control during dormancy entry and exit. Interestingly, both of these metabolic pathways are known to have extensive regulatory crosstalk with epitranscriptomic mechanisms in the same cells that they control dormancy (Li, Deng, et al. 2021; An and Duan 2022; Chen et al. 2024). Thus, post‐transcriptional control is not an isolated layer but is functionally integrated with upstream signaling and environmental sensing. Finally, other post‐transcriptional mechanisms playing important roles in dormancy such as microRNAs (Huo et al. 2016; Ruksha 2019; Roberts et al. 2024) and alternative splicing (Penfield et al. 2010; Li, Yan, et al. 2021; Singh and Ahi 2022) are tightly regulated by epitranscriptomic mechanisms such as m^6^A RNA modification (Erson‐Bensan and Begik 2017; Mei et al. 2023; Zhu et al. 2023) (Figure 1B).

## 4 The Reversible Nature of Dormancy and RNA Modifications

A hallmark feature of dormancy is its reversibility, the ability of cells or organisms to return to an active, proliferative, or developmental state upon receiving appropriate stimuli (Miller et al. 2021; Pshennikova and Voronina 2022; Özgüldez and Bulut‐Karslioğlu 2024). This reversibility distinguishes dormancy from terminal differentiation or senescence and underpins its adaptive value in fluctuating environments (Fujimaki and Yao 2020). In recent years, RNA modifications have emerged as prime candidates fulfilling these criteria, offering a versatile means of regulating gene expression without permanent genomic changes.

The best‐characterized RNA modification to date, N6‐methyladenosine (m6A), has been shown to influence a wide array of post‐transcriptional processes, including mRNA stability, splicing, nuclear export, and translation efficiency (Meyer and Jaffrey 2014; Meyer 2019). Importantly, m6A is installed by “writer” complexes such as METTL3/METTL14, removed by “eraser” enzymes like FTO and ALKBH5, and interpreted by “reader” proteins (e.g., YTH domain‐containing proteins) (Zaccara et al. 2019). This tripartite system enables dynamic and reversible control over RNA fate (Fu et al. 2014; Xiong et al. 2017), which is particularly advantageous in dormant cells that must remain in a poised but inactive state. The reversibility of RNA modifications mirrors the reversible entry and exit from dormancy observed across biological contexts. For instance, in hematopoietic stem cells, m6A levels are dynamically regulated during transitions between quiescent and active states, with specific m6A readers promoting the translation of cell cycle regulators upon activation (Wang et al. 2018; Yao et al. 2018; Chang et al. 2024). In dormancy contexts, changes in m6A status have been reported at multiple levels, including altered abundance of m6A writers/erasers/readers and selective remodeling of m6A‐marked transcript subsets. For example, prolonged quiescence in hematopoietic stem cells has been associated with reduced m6A‐dependent turnover of specific mRNAs following perturbation of m6A pathways, whereas in cancer models altered expression of m6A regulators (including elevated ALKBH5 in glioblastoma stem‐like cells) has been associated with dormancy‐linked gene expression programs (Cui et al. 2017; Zhang et al. 2017; Wang et al. 2018; Yao et al. 2018). Although such studies support a functional link between m6A pathway state and dormancy entry/maintenance, the upstream signals that regulate writer/eraser activities before dormancy onset remain incompletely resolved and are likely to be context‐dependent. This molecular flexibility is ideally suited to the demands of dormancy, where a rapid shift in cellular state must be achieved without de novo transcription. It has also been proposed that external cues, such as hypoxia, nutrient availability, or oxidative stress, can modulate the activity of RNA‐modifying enzymes, thereby linking the extracellular environment directly to RNA fate (Cayir et al. 2020; Ahi and Singh 2024; Ahi 2025). While increasing evidence indicates that epitranscriptomic regulation is responsive to diverse environmental stressors and exposures, the upstream mechanisms by which such cues are sensed and transduced into writer/eraser/reader regulation remain incompletely defined. Such coupling can be mediated by cue‐driven shifts in metabolic cofactor availability and signaling‐dependent post‐translational regulation or relocalization of writer/eraser/reader proteins, thereby translating environmental state into changes in RNA mark deposition and interpretation (Kim and Lee 2021; Lin et al. 2024). This positions epitranscriptomic machinery as a sensor–effector interface that transduces environmental signals into changes in the translational landscape, an essential capability for reversible dormancy (Ramnanan et al. 2009; Buijs et al. 2020). By harnessing the inherent flexibility of RNA chemical marks, cells are able to execute reversible gene expression programs that underpin survival, latency, and reactivation, traits that are evolutionarily selected and biologically indispensable. A brief mechanistic primer on writer–eraser–reader logic and cross‐taxa implementation is provided in Box 2.

BOX 2 **Writer–eraser–reader logic and cross‐taxa implementation.**

**Table:** 

**Writer–eraser–reader framework (using m6A as the exemplar mark)** **Writers** install marks on transcripts, creating the potential for state‐specific post‐transcriptional regulation. In animals, this is classically mediated by the METTL3/METTL14 complex; homologous writer systems occur across eukaryotes **Erasers** remove marks, enabling reversibility and rapid remodeling of marked transcript subsets (e.g., FTO/ALKBH5 in animals; ALKBH‐family demethylases in plants) **Readers** convert a chemical mark into an outcome by recruiting decay, translation, or localization machinery. Importantly, outcomes depend on which reader is engaged and where marks occur on the transcript, so the same mark can be associated with stabilization, decay, or enhanced translation in different contexts **Why stabilization vs. decay can flip** Marked transcripts can be routed toward different fates depending on the reader repertoire and cell state. For example, YTHDF‐family readers are frequently discussed in the context of decay/translation control, whereas other reader classes (e.g., IGF2BP proteins in mammals) can stabilize marked transcripts in some contexts—illustrating why mechanistic statements should specify the likely reader axis when possible **Beyond m6A (marks mentioned in this manuscript)—what “machinery” means** **m5C** is installed by NSUN‐family methyltransferases (e.g., NSUN2 in mammals; plant mRNA m5C mapping exists) and is frequently discussed in connection with RNA stability/processing **Pseudouridine (Ψ)** is installed by PUS enzymes and can alter translation properties of modified RNAs **Cross‐taxa snapshot: what is conserved vs lineage‐specific** **Plants:** m6A writers/erasers are conserved; YTH‐domain readers are represented by ECT‐family proteins, enabling transcript‐fate control in developmental transitions including germination‐related programs **Animals:** extensive mechanistic mapping exists for writers/erasers/readers; links to quiescence/dormancy‐like states often invoke reader‐dependent routing of marked transcripts into decay vs. translation/stabilization programs **Fungi:** m6A machinery is present and functional in developmental/stress‐linked states; reader‐mediated control is described (e.g., Pho92 in yeast), but dormancy–pathway coupling remains less fully charted than in animals/plants **Prokaryotes:** canonical eukaryotic m6A writer/eraser systems are not established; bacterial RNA modification biology is historically strongest for rRNA/tRNA enzymes, while mRNA epitranscriptome mapping is emerging and pathway‐level dormancy coupling is best treated as preliminary

## 5 Mechanistic Insights: Epitranscriptomic Marks That Modulate Translation, Stability, and Localization

RNA modifications have gained increasing recognition for their role in modulating the functional fate of transcripts. These modifications, which decorate coding and noncoding RNAs, have been observed to influence three major post‐transcriptional processes highly relevant to dormancy: mRNA stability (Basbouss‐Serhal et al. 2017; Vargas‐Blanco and Shell 2020), translation efficiency (Basbouss‐Serhal et al. 2015; Lorenzo‐Orts and Pauli 2024), and subcellular localization (Xia et al. 2019). Each of these regulatory dimensions contributes to how cells manage their protein output in states of low metabolic activity, making them particularly relevant to dormant conditions where selective protein synthesis is required.

Among the various known RNA modifications, m6A is the most extensively characterized in eukaryotic systems (Meyer and Jaffrey 2014; Meyer 2019). It has been shown to mark mRNAs for differential decay rates; for instance, methylation near the 3′ untranslated region can facilitate transcript degradation via recruitment of YTHDF2 (Sikorski et al. 2023). In contrast, methylation in coding sequences or near the 5′ UTR can enhance translation through recognition by other reader proteins, including YTHDF1 and YTHDC2 (Sikorski et al. 2023). This context‐dependent interpretation of RNA marks allows a single modification to produce opposing functional outcomes depending on its placement and associated readers (Shi et al. 2019; Pashay Ahi 2025).

Epitranscriptomic marks also control translation efficiency (Meyer 2019), which is especially critical when dormancy is accompanied by global downregulation of protein synthesis (Ramnanan et al. 2009; Buijs et al. 2020; Koli and Shetty 2024). Through direct modification of the mRNA or via reader‐mediated recruitment of translation machinery, these marks can determine which transcripts bypass translational repression. For example, specific m6A modifications have been associated with cap‐independent translation initiation, a mechanism that is favored under stress or when eIF4E‐mediated cap binding is inhibited (Meyer et al. 2015; Coots et al. 2017) (Figure 1B). This mechanism has been demonstrated under stress and cap‐inhibition conditions in mammalian systems and provides a concrete example of how selective translation can proceed when canonical cap‐dependent initiation is curtailed; whether and how cap‐independent initiation is deployed during dormancy transitions remains to be tested in dormancy‐specific models (Meyer et al. 2015; Coots et al. 2017).

In terms of localization, modifications like m^6^A and pseudouridine have been found to guide mRNAs into stress granules or P‐bodies; cytoplasmic sites involved in mRNA storage or decay (Vaidyanathan et al. 2017; Eyler et al. 2019; Fu and Zhuang 2020; Loedige et al. 2023) (Figure 1B). These compartments have been repeatedly observed in dormant or quiescent cells across different organisms (Davies et al. 2012; Zhang et al. 2013; Shah et al. 2014; Lee et al. 2016; Kearly et al. 2024; Koli and Shetty 2024). The inclusion or exclusion of mRNAs in these hubs depends, in part, on m^6^A status and its readers (e.g., YTHDF proteins) (Anders et al. 2018; Sun, Zuo, et al. 2023). Thus, m6A‐reader interactions act as sorting signals, triaging dormant mRNAs to storage/decay versus selective translation in a manner aligned with dormancy‐associated priorities.

Beyond m6A, other modifications such as 5‐methylcytosine (m5C) and pseudouridine (Ψ) are also gaining attention for their potential roles in dormancy (Blanco et al. 2011; David et al. 2017; Gkatza et al. 2019; Song and Wood 2020; Soto et al. 2022; Lorenzo‐Orts and Pauli 2024). m5C has been implicated in RNA export and stability, while pseudouridine is thought to influence RNA folding and translational fidelity (Wiener and Schwartz 2020). The full functional scope of these modifications in dormant states remains underexplored, though early findings suggest that they contribute to the precise tuning of RNA behavior required for long‐term survival. Their capacity to govern stability, translation, and localization in a selective manner makes them ideally suited to control gene expression during dormancy. These mechanisms provide a flexible yet specific mode of regulation that does not depend on ongoing transcription or permanent genetic changes, features that align closely with the core requirements of the dormant state.

## 6 Studies Linking Epitranscriptomics to Dormancy in Diverse Organisms

Across living organisms, diverse forms of dormancy, including seed and bud dormancy in plants, diapause in animals, quiescence in stem cells, sporulation and cyst formation in microbes, and hibernation or estivation in animals, represent distinct but functionally analogous survival strategies (Wilsterman et al. 2021; de Queiroz and Meyer 2023; Özgüldez and Bulut‐Karslioğlu 2024; Webster and Lennon 2025). Certain molecular mechanisms, such as RNA chemical modifications, are emerging as key candidates that may contribute to this convergent strategy across domains of life. Examples from multiple biological systems have begun to demonstrate a functional intersection between epitranscriptomics and dormancy. These case studies provide crucial validation of the hypothesis that RNA modifications may participate in regulating entry into, maintenance of, and exit from dormant states. Though much of the mechanistic detail remains under active investigation, current findings across plants (Tang et al. 2021; Wang, Zhang, et al. 2024; Li, Ma, et al. 2025), animals (Rehman et al. 2023; Wade et al. 2023), microbes (Antoine et al. 2021; Kouvela et al. 2021; Zhang, Zhang, et al. 2024), and cancer and stem cell biology (Blanco et al. 2011; Gkatza et al. 2019; Collignon et al. 2023; Zhang, Fu, et al. 2024) reveal regulatory patterns that support a potentially important role for epitranscriptomic control.

In plant biology, dormancy is most prominently observed in seeds, which must survive long periods in a metabolically inactive state (Kapás et al. 2024; Maleki et al. 2025). Recent transcriptome‐wide mapping in the plant model, *Arabidopsis thaliana* , has revealed dynamic changes in m6A methylation patterns during the activation of germination. Also, an m6A mRNA eraser (demethylase) ALKBH10B and an m6A reader ECT1 have been implicated in the transition from dormancy to germination in Arabidopsis (Tang et al. 2021; Li, Ma, et al. 2025). These modifications are correlated with altered stability and translatability of transcripts involved in hormone signaling, particularly abscisic acid and gibberellin pathways (Amara et al. 2022; Yin, Ao, et al. 2022; Li, Yin, et al. 2025; Shen and Yu 2025; Wang et al. 2025), which are known to regulate dormancy depth and release in plants (Zheng et al. 2015; Wang, Chen, et al. 2024). Moreover, a recent study has demonstrated the involvement of m6A RNA modification in regulation of bud dormancy in plants (Wang, Zhang, et al. 2024). Additional evidence has been reported in cereals: Participation of the m6A methyltransferase OsMTA1 in rice seed germination has been supported by transcriptome‐wide m6A profiling, with associated effects on germination‐ and stress‐responsive transcripts (Li, Yin, et al. 2025). These findings further support the view that dormancy‐to‐growth transitions in plants can be accompanied by remodeling of m6A‐marked mRNA populations. m6A marks were also enriched in transcripts associated with desiccation tolerance, suggesting a potential role in stress preparedness during dormancy (Han et al. 2023; Wu et al. 2024).

In the microbial world, *Mycobacterium tuberculosis* (Mtb) provides a compelling example of long‐term dormancy in the form of latent infection (Gengenbacher and Kaufmann 2012). During the latent phase, Mtb enters a nonreplicative but metabolically active state. Although much attention has been placed on transcriptional regulators such as DosR (Boon and Dick 2012), new evidence points to RNA‐based mechanisms as well. Pseudouridine and m6A modifications have been identified to play a role in mechanisms contributing to Mtb dormancy (Tomasi et al. 2023; Ma et al. 2024), with indications that they may influence the stability of stress–response transcripts under hypoxia or nutrient starvation, conditions characteristic of granulomatous dormancy. In dormant *Bacillus subtilis* spores, 5′‐NAD capping of mRNAs has been detected, supporting the presence of RNA modification‐associated transcript states in a canonical microbial dormancy system (Craft et al. 2020). Such modified mRNA species provide a concrete example of how RNA chemical marks may persist in dormancy and shape post‐transcriptional behavior during reactivation.

In animals, metabolic rate depression (MRD) is a unifying physiological state underlying various forms of dormancy, including hibernation, estivation, torpor, and diapause (Storey and Storey 2004; Staples 2016). Characterized by a profound, reversible reduction in energy consumption and biochemical activity, MRD enables animals to conserve resources, maintain cellular integrity, and survive prolonged periods of environmental stress such as cold, heat, or radiation (Gardner et al. 2025). Despite differing triggers and durations, these dormant states converge on MRD as a shared metabolic adaptation for endurance. Recent studies in animals revealed MRD‐related mechanisms involving m^6^A RNA modification such as hypoxia‐induced MRD condition in naked mole‐rats, *Heterocephalus glaber* (Ingelson‐Filpula et al. 2024), freezing and anoxia‐induced brain MRD in wood frogs, *Rana sylvatica* (Wade et al. 2023), and dehydration‐induced whole‐body MRD in the African clawed frog, *Xenopus laevis* (Rehman et al. 2023). During the diapause of bivoltine silkworm ( *Bombyx mori* ), a m6A reader has been shown to play a pivotal role in the regulation of the mRNA stability of genes in the ecdysone synthesis pathway, which are required for this process (Chen et al. 2022; Chen, Jiang, et al. 2023). Although intriguing, these examples highlight that our understanding of RNA modification–mediated mechanisms in animal dormancy remains in its early stages, with much still to uncover. They point to a promising frontier in organismal biology, where future research may reveal how epitranscriptomic regulation shapes dormancy across diverse animal systems.

In hematopoietic stem cells (HSCs), quiescence serves as a protective mechanism that preserves the long‐term regenerative capacity of the cell population. Several studies have shown that the m6A writer METTL3 is essential for HSC activation, while its depletion promotes prolonged quiescence and impairs hematopoietic recovery (Wang et al. 2018; Yao et al. 2018; Yin, Chang, et al. 2022; Zuo et al. 2024). Specific targets of m6A‐mediated regulation include mRNAs encoding cell cycle drivers and metabolic regulators. These findings suggest that RNA methylation contributes to the timing and coordination of dormancy exit, enabling a precise transition back to proliferation. In cancer biology, tumor cell dormancy represents a major clinical challenge due to its link to therapy resistance and metastatic relapse. RNA modifications have been found to be dysregulated in dormant cancer cells (Collignon et al. 2023). For instance, high expression of the demethylase ALKBH5 has been correlated with increased dormancy in glioblastoma stem‐like cells, partly through the stabilization of transcripts encoding quiescence‐associated transcription factors (Cui et al. 2017; Zhang et al. 2017). Other cancers, including breast and melanoma, show alterations in the balance of m6A writers and erasers during periods of therapeutic dormancy (Yang, Zhang, et al. 2022), suggesting that the epitranscriptome is actively remodeled to support survival without proliferation.

Each of these case studies points to a shared theme: The selective remodeling of RNA modifications is associated with transitions into and out of dormancy. Whether through controlling transcript decay in plants, translational priming in stem cells, or stress adaptation in pathogens and tumor cells, epitranscriptomic mechanisms appear to serve as regulatory switches that operate across a wide range of biological contexts.

## 7 Epitranscriptomics as a Fast, Flexible Toolkit for Adaptation

In fluctuating or hostile environments, dormancy allows cells and organisms to temporarily suspend growth while remaining viable (Jobava et al. 2021; Auld et al. 2023; Gianinetti 2023; Măgălie et al. 2023; Roberts et al. 2023; Starkloff et al. 2024; Nevermann et al. 2025). The regulatory systems supporting such plasticity are expected to operate efficiently under low‐energy conditions, respond quickly to environmental change, and remain evolutionarily adaptable. Within this context, epitranscriptomics acts as a regulatory system that fulfills these criteria. It functions as a post‐transcriptional toolkit that is both fast‐acting and evolutionarily flexible (Dannfald et al. 2021; Yang, Patil, et al. 2022; Ahi and Singh 2024). Unlike changes at the DNA or chromatin level, RNA modifications do not require permanent alterations to the genome. Instead, they enable rapid and reversible control of gene expression at the RNA level (Fu et al. 2014; Xiong et al. 2017; Leighton et al. 2018). From an evolutionary standpoint, RNA‐modifying enzymes and reader proteins are conserved across eukaryotic lineages. For example, homologs of METTL3 and FTO, the m6A writer and eraser enzymes, have been identified in plants, animals, and fungi (Sibbritt et al. 2013; Wong and Eirin‐Lopez 2021; Liu et al. 2022; Kim et al. 2024). Yet, despite this conservation, considerable functional diversification has occurred, allowing RNA modification pathways to be tailored to specific ecological niches and developmental programs (Liu et al. 2022; Wilkinson et al. 2022; Sun, Li, et al. 2023; Ahi et al. 2024; Frapin et al. 2026).

In microbial species, RNA modifications have been implicated in persistence‐related physiology and recovery dynamics, and these effects are consistent with a bet‐hedging framework in which phenotypic heterogeneity enhances survival under unpredictable threats; however, direct evidence that a specific RNA mark drives purely stochastic dormancy entry remains limited (Hou et al. 2020; Antoine et al. 2021; Morawska et al. 2022). In plants, seed dormancy has evolved in multiple lineages, often in response to local or global climatic pressures (Koutouan‐Kontchoi et al. 2020; Chen, Chen, et al. 2023; de Queiroz and Meyer 2023; Rosbakh et al. 2023; Jayasuriya and Phartyal 2024; Wang, Ge, et al. 2024; Jaganathan and Phartyal 2025). The ability to adjust the sensitivity of dormancy‐related transcripts through epitranscriptomic mechanisms may offer a tunable system that enhances fitness across diverse environments (Tognacca and Botto 2021; Xiang et al. 2024). In higher organisms, the evolutionary adaptation of epitranscriptomic systems has been associated with lifespan extension (McMahon et al. 2021; Wagner and Schosserer 2022), tissue regeneration (Weng et al. 2018; Cui et al. 2023), reproductive timing (Ahi et al. 2024; Frapin et al. 2026) and cancer resistance (Tang et al. 2024). These processes involve quiescent or dormant cellular states (Stuart and Brown 2006; Heyman et al. 2014; Rumman et al. 2015). For instance, long‐lived mammals exhibit distinct expression patterns of RNA‐modifying enzymes in tissues known to harbor dormant cells (e.g., skeletal muscles, brain, hair follicles, and bone marrow) (Ozkurede et al. 2019; Jiapaer et al. 2022; Yin, Chang, et al. 2022; Wu, Lu, et al. 2023; Ogbe et al. 2024).

RNA marks interact with stress pathways, metabolic sensors, and signaling cascades. They serve as modular units that integrate with pre‐existing systems without extensive genetic rewiring. This modularity may explain their frequent repurposing across taxa to regulate dormancy under diverse physiological and environmental conditions. Therefore, the epitranscriptome can be viewed as a core regulatory infrastructure that enhances the evolutionary adaptability of dormancy. It operates with speed, specificity, and minimal energetic demand, properties that are consistently favored under conditions where survival depends on reversible growth arrest and precise reactivation timing.

## 8 Toward a Unified Model: The Epitranscriptomic Regulation of the Dormant State

A unified conceptual framework is emerging, positioning the epitranscriptome as a central regulator of dormancy. In this unified model, RNA modifications function as key molecular signals that mediate the transition between active and dormant states, modulate transcript fate in response to environmental inputs, and support reactivation when conditions improve. Within this model, the initiation of dormancy involves both transcriptional and post‐transcriptional changes. As transcription slows, a subset of transcripts is selectively marked by modifications such as m6A, which either stabilize them for later use or direct them toward silencing in granules (Heck and Wilusz 2019; Alriquet et al. 2021; Collignon et al. 2023; Loedige et al. 2023; Zhang, Fu, et al. 2024). These decisions are governed by RNA‐binding proteins that recognize specific modifications and coordinate the recruitment of decay factors, translational machinery, or storage compartments (Loedige et al. 2023; Song et al. 2024; Zuo et al. 2024).

Maintenance of the dormant state is achieved through continued repression of translation, paired with selective access to pre‐existing transcripts that remain protected and responsive (Collignon et al. 2023; Li, Peng, et al. 2023; Lorenzo‐Orts and Pauli 2024). RNA marks serve as molecular bookmarks, allowing the cell to preserve information without active transcription. This preservation ensures that essential stress–response or metabolic genes can be re‐engaged quickly when conditions change, without the need for new RNA synthesis (Zhou et al. 2015). Upon exit from dormancy, RNA modifications are reinterpreted by shifts in the expression or activity of writer, eraser, or reader proteins. External signals such as nutrient availability or temperature change may influence enzyme localization, substrate affinity, or cofactor availability, leading to a rewiring of the RNA modification landscape (Zhou et al. 2015; Collignon et al. 2023; Li, Peng, et al. 2023; Lorenzo‐Orts and Pauli 2024). This transition permits a rapid and energy‐efficient ramp‐up of protein synthesis that is essential for re‐entering the cell cycle or resuming development.

To test this model, implementation of a time‐course in an inducible dormancy system that combines low‐input RNA‐modification mapping, reader‐specific CLIP, and polysome/ribosome profiling across entry and exit would track mark deposition, reader engagement, and translational output. The unified model also accommodates context‐specific variations, such as differences in which transcripts are modified or how modifications are interpreted. These variations arise from differences in tissue type, developmental stage, or organismal lineage but are underpinned by the same general principles of reversible, mark‐driven regulation. Importantly, the model supports integration with other regulatory layers, including chromatin state, transcription factors, and metabolic cues.

## 9 Open Questions and Future Directions

Progress in defining dormancy‐associated RNA‐modification landscapes is constrained by practical limitations in low‐input mapping because dormant samples often yield limited RNA. In addition, widely used enrichment‐based approaches can be sensitive to input amount, provide peak‐level rather than single‐nucleotide resolution, and introduce assay‐ and batch‐dependent biases that complicate quantitative comparisons across dormancy transitions. Recent developments are beginning to mitigate these constraints, including single‐cell strategies that resolve cell type–specific m6A landscapes from limited material (Tegowski et al. 2024) and nanopore direct RNA sequencing workflows that can jointly capture transcript isoforms, poly(A) features, and modification‐associated signatures, although sensitivity and modification calling remain active areas of development (Tan et al. 2024; Riquelme‐Barrios et al. 2025; Wang et al. 2025). While significant progress has been made in identifying RNA modifications and their potential roles in dormancy, several testable questions remain. To sharpen the future directions, three hypotheses are proposed:

Hypothesis 1 *Dormant states are characterized by distinct, predictive epitranscriptomic signatures*.

Hypothesis 2 *Dormancy transitions are driven by context‐dependent reader engagement that reprograms transcript fate*.

It is hypothesized that specific RNA‐modification patterns (e.g., mark location and stoichiometry on key transcripts) distinguish dormancy depth and predict reactivation potential. This can be tested using low‐input and single‐cell RNA modification profiling across dormancy entry, maintenance, and exit, coupled to transcript abundance and translational state (Tegowski et al. 2024; Ahi and Khorshid 2025; Khorshid and Ahi 2025).

It is hypothesized that specific RNA‐modification patterns (e.g., mark location and stoichiometry on key transcripts) distinguish dormancy depth and predict reactivation potential. This can be tested using low‐input and single‐cell RNA modification profiling across dormancy entry, maintenance, and exit, coupled to transcript abundance and translational state (Tegowski et al. 2024; Ahi and Khorshid 2025; Khorshid and Ahi 2025).

Hypothesis 3 *Environmental and metabolic cues rapidly rewire the epitranscriptome by modulating writer/eraser activity*.

It is hypothesized that changes in reader availability, localization, or modification state shift the interpretation of identical RNA marks toward storage/decay versus selective translation, thereby gating dormancy maintenance and exit. This can be tested with reader‐specific CLIP (and complementary granule association assays) performed across dormancy entry/exit time‐courses, together with targeted perturbation of reader abundance or binding capacity (Nguyen and Kang 2024).

It is hypothesized that changes in reader availability, localization, or modification state shift the interpretation of identical RNA marks toward storage/decay versus selective translation, thereby gating dormancy maintenance and exit. This can be tested with reader‐specific CLIP (and complementary granule association assays) performed across dormancy entry/exit time‐courses, together with targeted perturbation of reader abundance or binding capacity (Nguyen and Kang 2024).

It is hypothesized that nutrient, hypoxia, temperature, and oxidative cues alter the activity or localization of writer/eraser enzymes, producing rapid remodeling of RNA marks that enables reversible dormancy transitions. This can be tested using inducible dormancy systems with entry/exit time‐courses combining low‐input modification mapping with enzyme perturbation and matched readouts of translational output (e.g., polysome/ribosome profiling) (Zhou et al. 2015; Cayir et al. 2020; Ingelson‐Filpula et al. 2024).

Beyond these hypotheses, additional layers of dormancy regulation are likely to intersect with RNA modifications. For instance, the extent to which RNA modifications interact with other novel emerging mechanisms of dormancy state regulation in various organisms and cells, such as small or long noncoding RNAs (Reynolds 2019), enhancer RNAs (So et al. 2022; Tremblay et al. 2024), RNA G‐quadruplexes structuring (Zuurbier et al. 2024), intracellular phase separation processes (Xu et al. 2021; Liu, Zhu, and Zhao 2023), and codon usage bias (Kanduc 2021; Small‐Saunders et al. 2024; Feng et al. 2025), is still unclear. Interestingly, all of these novel players in regulation of dormancy are known to have extensive regulatory crosstalk with m6A RNA modifications (Figure 1B). The role of feedback loops, where modifications influence the expression of their own modifying enzymes, also deserves further study, as such loops could stabilize or destabilize dormancy states at cellular level (Yeo et al. 2018; Deritei et al. 2019; Wu, Sun, et al. 2023). In fungi, where current evidence is preliminary, future work should prioritize mechanistic dissection of m^6^A–pathway crosstalk (e.g., TOR/PKA/AMPK) using low‐input mapping and perturbation experiments across sporulation and quiescence entry/exit. Finally, the therapeutic implications of modulating RNA modifications in dormant cells remain largely unexplored. In cancer, targeting the epitranscriptome could potentially force dormant cells into reactivation and subsequent vulnerability to therapy (Tamamouna et al. 2022). In agriculture, manipulating RNA modification patterns in seeds might offer strategies for improving crop resilience or germination control (Lieberman‐Lazarovich et al. 2022).

## Author Contributions

**Ehsan Pashay Ahi:** conceptualization (lead), investigation (lead), project administration (lead), validation (lead), visualization (lead), writing – original draft (lead), writing – review and editing (lead).

## Funding

The author has nothing to report.

## Ethics Statement

The author has nothing to report.

## Conflicts of Interest

The author declares no conflicts of interest.

## Data Availability

Data sharing is not applicable to this article as no datasets were generated or analyzed during the current study.
